# The Developmental Toxicity of *Thymus schimperi* Essential Oil in Rat Embryos and Fetuses

**DOI:** 10.1155/2022/4091839

**Published:** 2022-04-11

**Authors:** Fentahun Adane, Kaleab Asres, Wondwossen Ergete, Samuel Woldekidan, Girma Seyoum

**Affiliations:** ^**1**^ Department of Anatomy, School of Medicine, Debre Markos University, Debre Markos, Ethiopia; ^2^Department of Pharmaceutical Chemistry & Pharmacognosy, College of Health Sciences, Addis Ababa University, Addis Ababa, Ethiopia; ^3^Department of Pathology, School of Medicine, College of Health Sciences, Addis Ababa University, Addis Ababa, Ethiopia; ^4^Ethiopian Public Health Institute, Biomedical and Clinical Research Team, Traditional and Modern Medicine Research Directorate, P.O. Box 1242, Addis Ababa, Ethiopia; ^5^Department of Anatomy, College of Health Sciences, Addis Ababa University, Addis Ababa, Ethiopia

## Abstract

**Background:**

In Ethiopian traditional medicine, the aerial parts of *Thymus schimperi* are widely used to treat diseases such as gonorrhea, cough, liver disease, kidney disease, hypertension, stomach pain, and fungal skin infections. In addition, they have been used as vegetables to flavor a broad variety of food products. However, there is an insufficient investigation of the toxic effect of *Thymus schimperi* essential oil. The aim of this study was, therefore, to evaluate the developmental toxicity of the essential oil of *Thymus schimperi* leaves on developing rat embryos and fetuses.

**Methods:**

Essential oil of the aerial parts of *Thymus schimperi* was extracted by hydrodistillation. Pregnant Wistar albino rats were randomly divided into five groups. The doses 65 mg/kg, 130 mg/kg, and 260 mg/kg of the essential of *Thymus schimperi* were administered by force feeding to the III–V groups, respectively. Groups I and II were negative and *ad libitum* control groups. The embryos and fetuses were revealed on days 12 and 20 of gestations, respectively. The embryos were examined for developmental delays or growth retardation. Gross external, skeletal, and visceral anomalies in the fetuses were examined.

**Results:**

In this study, the developmental scores of the number of implantation sites, crown-rump length, the number of somites, and morphological scores were significantly lower while the score of fetal resorptions was increased in a 12-day-old rat embryos treated with 260 mg/kg of the *Thymus schimperi* essential oil. There was also a significant delay in the development of the otic system, olfactory system, and a reduction in the number of branchial bars in 12-day-old embryos treated with 130 mg/kg and 260 mg/kg of the essential oil. However, external morphological examinations of rat fetuses revealed no detectable structural abnormalities. The fetal skull, vertebrae, hyoid, forelimb, and hindlimb ossification centers did not differ significantly across all the groups. Furthermore, there were no skeletal or soft-tissue malformations as a result of the essential oil treatment. Although the difference was not statistically significant, fetuses of the high-dose treatment group had a reduced number of ossification centers in the caudal vertebrae and hind limp phalanges.

**Conclusion:**

The essential oil of *Thymus schimperi* at high doses has a detrimental effect on the development of rat embryos and fetuses. Its developmental toxicity is evidenced by significant delays in fetal and embryonic development, a decrease in the number of implantation sites, and an increase in fetal resorption. Furthermore, administration of the essential oil in higher doses resulted in a significant decrease in placenta weight and litter weight. In addition, the present study provided evidence that using the *Thymus schimperi* essential oil in a high dose could affect the developing embryo and fetus. Thus, it is recommended to discourage the use of *Thymus schimperi* essential oil in high doses.

## 1. Introduction

*Thymus schimperi* is known as “Tosign” in Ethiopia, and the leaves are used as flavors in a variety of food products and traditional medicines [1]. The fresh or dried leaves of *T. schimperi* are used locally as condiments and tea [1], in the preparation of “berbere” (pepper powder) and “shirro” (bean/pea powder) [2], and for the preparation of Metata ayb (fermented cottage cheese) [3]. In traditional medicines, *T. schimperi* is used to treat different illnesses like gonorrhea, cough and liver disease, renal diseases, hypertension [1], stomach pain [4], kidney problems [5], and dermal fungi [6].

*T. schimperi* consists of approximately 1.0% to 2.5% of volatile oil [7]. Essential oils are volatile, natural, and complex chemicals with a strong odor that are synthesized as secondary metabolites by aromatic plants. Steam or hydroxdistillation is commonly used to obtain essential oils [8, 9]. There are about 3000 essential oils known today, with 300 of them being commercially relevant, particularly in the medicinal, agronomic, food, sanitary, cosmetic, and perfume industries [10].

The composition of the volatile oil varies depending on the chemotype of the plants [11]. The principal components of *T. schimperi* essential oil are thymol and carvacrol (up to 64% of oil), along with linalool, p-cymol, cymene, thymine, a-pinene, Apigenin, luteolin, and 6 hydroxyluteolin glycosides, as well as di-, tri-, and tetra-methoxylated flavones have also been detected in *T. schimperi* leaves [7].

Although *T. schimperi* essential oil has antimicrobial activities [12], the leaves of *T. schimperi* are commonly used by locals as a food preservative, medicine for various ailments, and food flavoring and seasoning [13]. Pregnant mothers in Ethiopia are using *Thymus schimperi* by tea. Essential oil constitutes the plants is commonly used when fresh herbs are boiled in tea. However, no research has been done on the developmental toxicity of the *Thymus schimperi* essential oil. Therefore, this study aimed to assess the toxicity of *Thymus schimperi* essential oil in rat embryos and fetuses.

## 2. Materials and Methods

### 2.1. Plant Material Collection and Extraction

Fresh leaves of *T. schimperi* were collected from their natural habitats in March 2019 from Densho (Bale Mountains National Park, Oromia Region), which is 370 km southeast of Addis Ababa. The botanical identity of the plant was confirmed by the herbarium of the Ethiopian Public Health Institute (EPHI), where a voucher specimen (collection no. HHx001) was deposited for reference.

### 2.2. Essential Oil Extraction

Fresh leaves of *T. schimperi* (1 kg) were extracted by hydrodistillation using a Clevenger-type apparatus [14]. The oil obtained was stored in a sealed amber-coloured vial in a refrigerator at −10°C until it is used for the study [15].

### 2.3. Selection and Preparation of Experimental Animals

Wistar albino rats, 8 to 10 weeks of age, obtained from the Ethiopian Public Health Institute (EPHI) breeding unit, were used. The female rats were nulliparous and nonpregnant. Same-sex rats were acclimatized in a standard cage with five animals per group (*n* = 5) and held under standard conditions (at a temperature of 20°C (±2°C), with a normal 12-hour light/12-hour dark cycle). All the experiments were conducted following the internationally accepted laboratory animal use and care guidelines [16]. Animals were acclimatized for one week before the commencement of the study and were provided with water and food pellets *ad libitum* before and until the end of the experimental period [16].

After adaptation, the animals used for developmental toxicity studies were mated overnight by placing a male Wistar albino rat in a cage containing one nulliparous female rat. The male rat was introduced to the cage at approximately 17:00 hours. After overnight mating, female rats were inspected for the presence of copulatory plugs the following morning and vaginal smears were taken for microscopic determination of the presence of sperm. The presence of spermatozoa in the vaginal examination was considered as a 0 day of gestation [16].

### 2.4. Grouping and Dosing of Animals

Pregnant Wistar albino rats were randomly divided into five groups, each comprising 10 pregnant rats. Two control groups were a pair-fed control group (Group I) and an *ad libitum* control group (Group II). The pair-fed control group received the vehicle (distilled water and 2% tween 80) and the same amount of food and water as the experimental groups while the *ad libitum* control group was untouched and fed *ad libitum*. The experimental groups (Groups III–V) were given 65 mg/kg, 130 mg/kg, and 260 mg/kg of vehicle dissolved essential oil of *T. schimperi*. The extract was weighed and mixed with a vehicle (distilled water and 2% tween 80) and continuously vortexed with a vortex shaker. The final volume was 2 ml/100 g with the vehicle, and the oral gavage was used for oral administration [17].

### 2.5. Developmental Toxic Effects of the Essential Oil

#### 2.5.1. 12-Day Experiment

This experiment was developed to assess the potential developmental toxicity of the essential oil extracts of *T. schimperi* on pregnant animals in 12-day-old whole rat embryos. The experiment was intended to expose any growth and developmental abnormalities that may not have been noticeable in the near term fetuses due to potential compensatory growth and development. After pregnancy was verified, pregnant rats were randomly assigned to control groups, Group I (pair-fed control) and Group II (*ad libitum* control), and treatment groups, Group III (EOT 65 mg/kg), Group IV (EOT 130 mg/kg), and Group V (EOT 260 mg/kg), respectively.

Each group consists of ten pregnant rats. Group I (pair-fed control) received the vehicle (distilled water with 2% of tween 80), and Group II was untouched/unrestricted-fed (*ad libitum* control) while Group III, Group IV, and Group V were received the vehicle dissolved essential oil 65 mg/kg/day, 130 mg/kg/day, and 260 mg/kg/day, respectively. The treatment period was from days 6 to 12 of gestation. The reason for treatment administration from day 6 through day 12 of gestation lies in the fact that this time represents an active embryogenesis and organogenesis period. The critical developmental period in the rat is embryonic days 6 to 12 [16].

The gravid rats were euthanized by intraperitoneal injection of pentobarbital (150 mg/kg of body weight) [18] at the end of the treatment period (day 12 of gestation). The uterine horns were removed and placed in Hank's balanced salt solution. They were then incised along the border of the antimesometrium to expose the embryos. The membranes covering the embryo were separated with the aid of a fine pair of forceps and a dissecting microscope to expose the underlying visceral yolk sac. Circulation and growth of the yolk sac were assessed. The embryos were then explanted, and the development of the circulatory, nervous, visual, auditory, olfactory, and skeletal systems, as well as craniofacial development, was assessed quantitatively based on 16 recognizable developmental end points (morphological scores), according to the criteria of Brown and Fabro [19]. The numbers of somites have also been counted.

#### 2.5.2. 20-Day Experiment

This experiment was intended to determine the potential developmental toxic effects of the *T. schimperi* essential oil on near term fetuses. Once pregnancy was confirmed, animals were also randomly allocated to control groups, Group I (CON) and Group II (*ad libitum* control group), and treatment groups, Group III (EOT 65 mg/kg), Group IV (EOT mg/kg 130), and Group V (EOT 260 mg/kg). Each group consists of ten pregnant rats. The vehicle was provided to the control Group I (CON). Group II was unregulated *ad libitum*. The treatment groups (Group III, IV, and V) were received 65 mg/kg/, 130 mg/kg, and 260 mg/kg/day vehicle dissolved essential oil of *T. schimperi*, respectively.

A control diet of an equal amount was provided for each animal in the experimental groups (Group III, IV, and V), and the control group was given the same diet and kept in the same setting except essential oil given only for the experimental groups. Every morning, every animal's daily food intake was recorded, animals were weighed, and weight gain was recorded on days 1, 6, 12, and 20 of gestation [16].

Gravid females were anesthetized on a gestational day 20 by intraperitoneal injection of pentobarbital (150 mg/kg of body weight) [18]; the uterine horns were exposed and examined intact. The number of implantation sites was determined by counting the metrial glands situated along the mesometrial margin of the uterine horns, which are yellowish nodules. The number of prior resorptions was measured by metrial nodules, which were not occupied by living or recently dead fetuses. Gentle pressure on them was exerted to assess the amount of live or dead fetuses. To expose the fetuses, fetal membranes, and the placenta, the uterine horns were incised along the antimesometrial border. The fetuses were then retrieved and placenta-free dissected. Following these measurements, the length of the crown-rump (CRL) and the placental weight was recorded. Fetuses were fixed for visceral examination and gross external inspection in Bouin's solution (aqueous saturated solution of picric acid 75% t, formalin 25%, and glacial acetic acid 5%) [16].

### 2.6. Visceral Examination

Following an external examination of the fetuses at necropsy, additional soft-tissue examination by serial sectioning was performed. For two weeks, the fetuses were fixed in Bouin's solution and serially sectioned. The sectioning was performed using a surgical blade and the Modified Wilson technique [20]. Under a dissecting microscope, sections were taken craniocaudally at 1–2 mm intervals (XTL3101, 6*x* magnification). The first section was made through the jaw and passed posteriorly above the ear. The palate was examined for the presence of any clefts after the tongue was removed. A coronal section of the head was performed as well as a transverse section of the neck and parts below. The following organs were examined for any observable anomalies: brain (hydrocephalus, dilation of ventricles, and microphthalmia/anophthalmia), craniofacial region (nasal septum defect and cleft palate), thoracic region (lungs: lobar defect and heart: septal defect and retro esophageal aortic arch), abdominal region (liver, stomach, and gut anomalies), and pelvic region (kidneys: agenesis, ectopic kidney, and hydronephrosis and gonads: testes and ovarian anomalies).

### 2.7. External Evaluation

Fetuses fixed in Bouin's solution were checked head to tail for gross external malformations under a dissecting microscope for day 20 experiment [21]. The criteria to be evaluated were as follows:

- Craniofacial development (exencephaly, anencephaly, microphthalmia, and anophthalmia)
- Development of the limbs (syndactyly, adactyly, and polydactyly)
- The vertebral column (neural tube defect, kyphosis, and scoliosis)
- Tail development (missing tail)
- External genitalia

### 2.8. Skeletal Staining

Skeletal staining was performed using the method of Young et al. [22]. Depending on the size of the litter, 2 or 3 fetuses per litter have been completely eviscerated by a small midline incision in the anterior abdominal wall. Eviscerated fetuses (2 or 3) were then put in a small bottle of 95% alcohol and dehydrated for a minimum of one week. After dehydration, the specimens were cleared with 1% potassium hydroxide in a solution until the bone was cleared normally for two days. The specimens were then moved to the new 1% potassium hydroxide (KOH) solution and stained with a few drops of alizarin red (0.4 ml). The staining continued overnight, and overstraining was fixed by storing the samples in the solution of the Mall (79% distilled water, 20% glycerin, and 1% KOH). Increasing glycerin concentrations (20%, 40%, 60%, and 80%) were then passed through the specimens for about one week in each concentration and finally stored for evaluation in l00% glycerin. To avoid fungal growth and contamination when stored in pure glycerin, a small thymol crystal was added.

### 2.9. Skeletal Evaluation

Using a skeletal scoring chart, developed by Nash and Persaud [23], which is a modification of the scoring system stated by Aliverti et al. [24], the skeletal assessment was performed. Under the microscope, ossification of the hyoid, sternebrae, metacarpal, metatarsal, and thoracic bones were studied and the number of ossified centers was counted. The primary indices of skeletal development in the rat were stated as the degree of ossification of the sternebrae, metacarpal, metatarsal, and sacrococcygeal bones.

### 2.10. Data Processing and Analysis

Data were entered using EpiData version 3.02 and was exported to SPSS version 24 for analysis. All data represented by numbers were analyzed by SPSS statistical software. All values have been expressed in mean ± SDM (standard deviation of the mean). Treatments over time were compared by using one-way analysis of variance (ANOVA) between control and treated groups followed by Dunnett's *t* test to determine the significance level. The data regarding embryonic development were analyzed by using chi-square analysis. Statistically significant differences at *p* < 0.05 were considered.

### 2.11. Ethical Consideration

In this study, the Institutional Review Boards of the College of Health Sciences (IRBCHS), Addis Ababa University with a protocol number of 036/2018/anat and form AAUMF 03-008. The use of animals and all activities in this experimental study were conducted following the AAUMF regulations for animal care and use.

## 3. Results

### 3.1. Day 12 Experiment

#### 3.1.1. Pregnancy Outcomes

**Figure 1:**
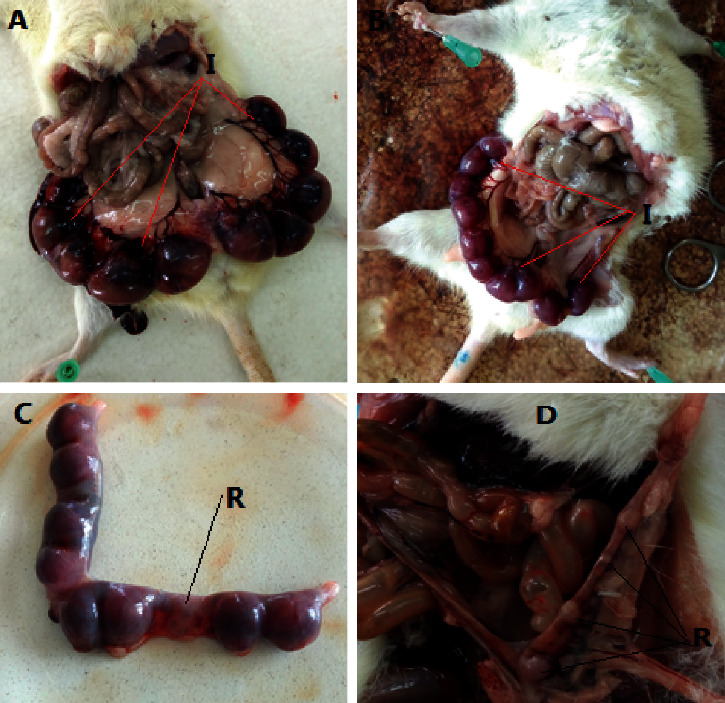
Number of implantation and resorption sites: (a) (*ad libitum* control), (b) (65 mg/kg), (c) (130 mg/kg), and (d) (260 mg/kg). I, implantation site; R, resorption site.

In the current study, the maternal weight gain was significantly lower in a high-dose treatment group as compared to *ad libitum* and pair-fed control groups. In the high-dose treatment group and the *ad libitum* control group, the mean maternal weight gains were 1.73 ± 0.411 g and 3.10 ± 0.211 g, respectively. Similarly, there was a high incidence of fetal resorptions at a dose of 260 mg/kg as compared to the control groups. At a high-dose treatment group (260 mg/kg) and *ad libitum* control group, the fetal resorptions were 1.01 ± 0.611 and 0.35 ± 0.45, respectively. In addition, the number of implantation sites was significantly decreased at a high dose (260 mg/kg) (Table 1 and Figure 1).

#### 3.1.2. Growth of the Embryo

The crown-rump length (CRL) of rat embryos treated with 260 mg/kg of essential oil was significantly lower as compared to the pair-fed and *ad libitum* control groups. The mean CRL for the pair-fed and *ad libitum* control groups were 5.0 ± 0.6 and 5.1 ± 0.4, respectively, while the mean CRL for the high-dose (260 mg/kg) treatment group was 4.30.7. Similarly, the high-dose (260 mg/kg) group had a significantly lower mean number of somites than the control groups. Furthermore, rats given 260 mg/kg essential oil had a mean morphological score of 44.0 ± 0.5. The scores for the pair-fed and *ad libitum* control groups were 46.1 ± 0.2 and 45.8 ± 0.3, respectively. The mean morphological score in pregnant rats given 260 mg/kg *T. schimperi* essential oil was significantly lower than the control groups (Table 2).

#### 3.1.3. Embryonic Body System Development

**Figure 2:**
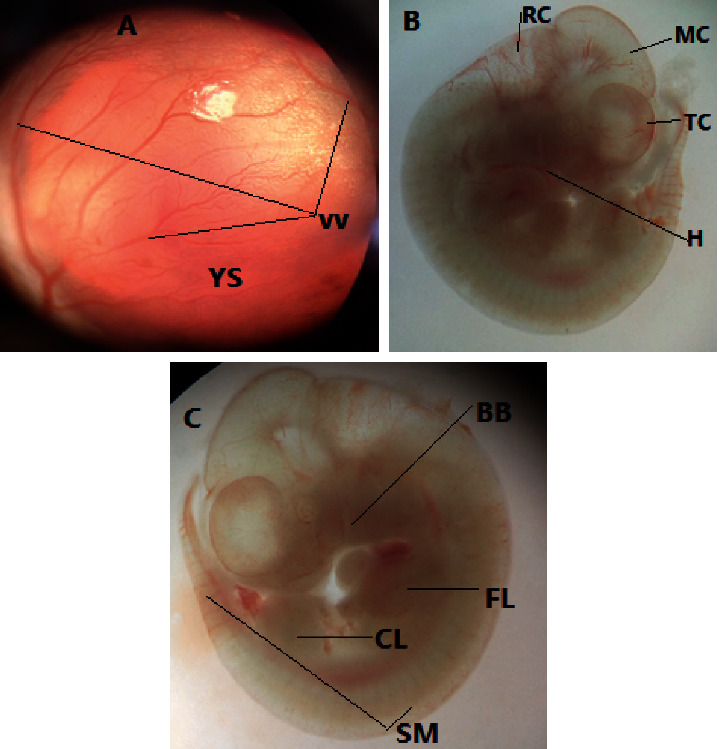
A 12-day-old rat embryos showing various organs of primordia. (a) An embryo enclosed inside the intact yolk sac (YS) with vitelline vasculature surrounding it (VV); (b) yolk sac-free embryo, separated from the surrounding blood vessels, revealing the heart (H), telencephalon (TC), mesencephalon (MS), and rhombencephalon (RC); and (c) brachial bars (BB), forelimb bud (FL), somites (SM), and caudal limb bud (CL).

The developmental parameters of the otic system, olfactory system, and branchial bars were changed significantly (Table 3 and Figure 2). The growth score of the otic system of rat embryos in the middle dose (130 mg/kg) and high dose (260 mg/kg) treatment groups of the essential oil was significantly lower as compared to the pair-fed and *ad libitum* control groups. Pair-fed and *ad libitum* control groups had a mean growth of 3.6 ± 0.51 and 3.5 ± 0.50, respectively. However, the score in the middle-dose (130 mg/kg) and high-dose (260 mg/kg) treatment groups were 3.1 ± 0.41 and 3.0 ± 0.40, respectively. Similarly, the olfactory system growth score of rat embryos from the middle dose (130 mg/kg) and high dose (260 mg/kg) were significantly lower than that of the pair-fed and *ad libitum* control groups. The scores were 0.9 ± 0.70 and 0.8 ± 0.62 in the pair-fed and *ad libitum* control groups, respectively. However, the scores in the middle-dose and high-dose treatment groups were 0.7 ± 0.60 and 0.5 ± 0.42, respectively. In addition, the high-dose group (260 mg/kg) had a significantly lower mean number of branchial bars (2.9 ± 0.33) as compared to the pair-fed (3.6 ± 0.55) and *ad libitum* control groups (3.5 ± 0.52). However, there was no significant difference in the yolk sac circulation, embryo flexion, heart, caudal neural tube, hindbrain, midbrain, forebrain, optic system, maxillary process, mandibular process, forelimb, and hind limb developmental parameters between the treatment and control groups.

### 3.2. Day 20 Experiment

#### 3.2.1. Pregnancy Outcomes

In the pretreatment (days 1–5), treatment (days 6–12), and posttreatment (days 13–20), there was no significant difference in maternal food intake between the treatment and control groups (Table 4).

In the present study, the maternal weight gain in the high-dose (260 mg/kg) group was 3.32 ± 0.56 g/week and 9.41 ± 0.32 g/week during the treatment (day 6–12) and posttreatment (day 13–20) periods, respectively. The pair-fed and the *ad libitum* control groups' maternal weight gain were 5.96 ± 0.61 g/week and 16.01 ± 0.75 g/week, respectively. During treatment (day 6–12) and posttreatment (day 13–20) periods, the maternal weight gain in the high-dose (260 mg/kg) treatment group was significantly lower than in the pair-fed and *ad libitum* control groups (Table 4).

In the current study, the fetal resorption of the high-dose (260 mg/kg) treatment group was significantly increased as compared to the control groups. In the dose of 260 mg/kg, the mean fetal resorption was 0.70 ± 0.18. However, the mean number of fetal resorptions was 0.20 ± 0.50 and 0.30 ± 0.52 in the pair-fed and *ad libitum* control groups, respectively.

In this study, the number of fetuses, and live fetuses, was lower in a dose-dependent manner across groups, but the differences were not statistically significant. Furthermore, the high-dose (260 mg/kg) treatment group had a higher fetal death rate than the other groups. However, it was not statistically significant (Table 5).

#### 3.2.2. Fetal Growth

In the current study, the high-dose treatment group's mean litter weight (2.71 ± 0.11) was significantly lower than the pair-fed control group (3.41 ± 0.09 g) and *ad libitum* (3.38 ± 0.10 g) control group. Furthermore, the mean growth score of the CRL was significantly lower in the high-dose treatment group (2.91 ± 0.18) than the pair-fed control (3.18 ± 0.11) and the *ad libitum* control groups (3.18 ± 0.11).

Similarly, the placental weight was significantly lower in the high-dose treatment group (0.48 ± 0.05 g) than the pair-fed control (0.55 ± 0.05) and the *ad libitum* control groups (0.57 ± 0.06) (Table 6).

#### 3.2.3. External Morphological Anomalies

**Figure 3:**
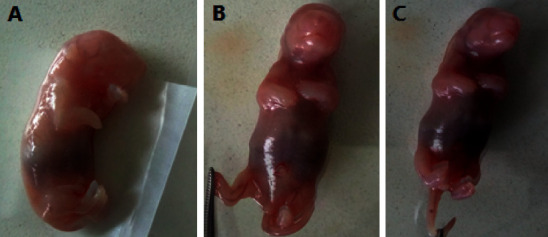
Live rat fetuses from high-dose rat groups: (a) fetus of the pair-fed control group, (b) fetus of the 130 mg/kg group, and (c) fetus of the 260 mg/kg dose group.

The explanted fetuses were also examined for external malformations from head to tail in the essential oil developmental toxicity study. Craniofacial abnormalities, limb defects, vertebral column anomalies, missing tails, and external genital abnormalities were all investigated. Despite this, there were no treatment-related defects in near term rat fetuses (Table 7 and Figure 3).

#### 3.2.4. Visceral Soft-Tissue Anomalies

**Figure 4:**
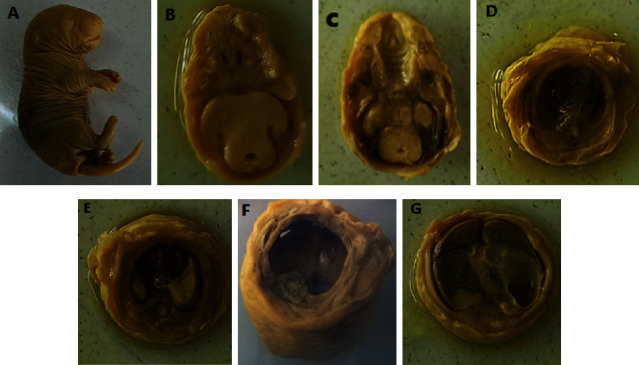
A 20-day-old rat fetus fixed in Bouin's solution for visceral examination (260 mg/kg). (a) Unsectioned fetus; (b) transverse section of the brain showing normal brain tissue; (c) normal palate; (d) a section made through the neck showing the normal esophagus, trachea, and thyroid gland; (e) a section made through the chest showing normal; (f) intact diaphragm; and (g) a section made through the abdomen showing normal visceral organs.

After being fixed in Boiun's solution, the fetuses were serially sectioned for visceral soft-tissue analysis. Serial sectioning was conducted on the head, neck, stomach, and abdomen. The parts were carefully examined under a dissecting microscope for any visceral abnormalities. The head area was investigated for cleft palate, hydrocephalus, and eye-related abnormalities. Thyroid glands, thymus, trachea, and cardiac septum abnormalities were also studied at the neck and chest stages. Diaphragmatic hernia, abdominal viscera agenesis, and external genitalia were also investigated. During the external morphological examination, no noticeable visceral abnormalities were identified (Table 8 and Figure 4).

#### 3.2.5. Skeletal Malformations

**Figure 5:**
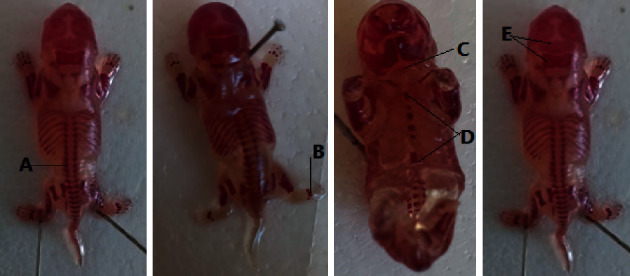
Alizarin red-stained rat fetuses showing different ossification centers. A: vertebrae, B: metatarsal, C: hyoid, D: sternebra, and E: supraoccipital and interparietal.

The developmental status of the skull, thoracic vertebrae, sternum, hyoid, and metatarsals was examined in this study, but no significant skeletal anomalies were found in either the treatment or control groups. However, the ossification centers of the caudal/coccygeal vertebrae and the hind limb phalanges differed slightly. In the high-dose (260 mg/kg) group of rat fetuses, the number of ossifications in the hind limb phalanges and caudal vertebrae was slightly reduced. It was, however, not statistically significant. There were developmental delays in the hindlimb phalanges in 26% of the rat fetuses from the high-dose group (260 mg/kg). However, the variation was not statistically significant. The skeletal analysis results are presented in Tables 9 and 10 and Figure 5.

## 4. Discussion

Although some biologically active substances found in medicinal plants are teratogenic, the vast majority of them have often been used in various forms in the community [25]. Toxic agents can either directly affect the embryo/fetus or indirectly affect the mother and enter the embryo/fetus via the placenta or they can be a combination of both [26]. The essential oil of *T. schimperi* has not been studied for its developmental toxic effects, despite the fact that the plant is widely used as a spice and has traditional medical uses due to its essential oil content.

The essential oil of fresh *T. schimperi* leaves obtained by hydrodistillation yielded 1.39% (w/w) yield. Carvacrol (49.90%), thymol (10.64%), o-cymene (8.54%), terpinene (4.5%), linalool (2.51%), and 3-octanol (2.48%) were the major constituents of the oil. In a recently published article in Ethiopia, the phytochemical analysis and toxicity profiles of the compounds in *Thymus schimperi* essential oil were stated [15].

In this study, rats were given different doses of the essential oil of *T. schimperi* to evaluate its developmental toxicity. The essential oil was given on days 6–12 of pregnancy, which is a critical period for embryogenesis. The skeletal system, soft-tissue growth, and the toxicity of rat embryos and fetuses were used to assess the developmental toxicity potential of the essential oil.

In the 20-day experiment, maternal weight gain was significantly reduced in a dose-dependent manner in the treatment and posttreatment periods. This finding is in line with a previous study by Abebe et al., in 2021, which found that *T. schimperi* inhibits weight gain in experimental rats [27]. The principal components of the *T. schimperi*, carvacrol, and thymol could be a possible explanation for this finding. Dietary carvacrol and thymol were reported to inhibit weight gain in animals via affecting lipid metabolism, with carvacrol having the ability to lower plasma triglycerides [28].

In the current study, treatment groups exposed to high doses of the essential oil had a higher incidence of fetal resorptions than the control groups. Because this study was conducted with crude essential oil, it is difficult to determine which bioactive compound increases the resorption number. However, the increment could be due to the effects of *T. schimperi* major phytochemicals, such as carvacrol and thymol which could cause cell cycle arrest in the G0/G1 phase, cellular apoptosis, and inhibition of cell proliferation [29, 30]. All these are the basic functions of cells for zygote growth. Similarly, the number of implantation sites in the high-dose treatment group of the essential oil extract of *T. schimperi* was significantly lower than the control groups. This result is comparable to a previous report by Prakash et al., who noted that treating rats with some native plants has an anti-implantation activity [31]. The disruption of hormone secretion, which plays an important role in embryo implantation, could be influenced by monoterpenoids components of essential oils, particularly carvacrol, which can disrupt progesterone secretion by changing the neurochemical and neurobehavioral profiles of rats [32]. Another possible explanation is that carvacrol, one of the most important essential oil constituents, inhibits prostaglandin synthesis [33]. This is crucial for the embryo's interaction with the endometrium such as implantation (apposition, adhesion/attachment, and invasion/penetration) and decidualization [34].

The number of somites, morphological score, litter weight, and CRL were evaluated to assess the growth of embryos and fetuses. Morphological scores are used to predict the embryo's growth in the in vivo study since they have a linear relationship with embryonic age [35]. In the current study, all these parameters were significantly lower at the high doses of the essential oil extract as compared to the control groups. In line with this study, studies conducted in Ethiopia found that high doses of *Syzygium guineense* (Myrtaceae), *Moringa stenopetala*, and *Acrylamide* were significantly lower the number of somites, morphological score, litter weight, and CRL of rat embryos and fetuses as compared to the controls [36–38]. This could be because the plants contain alkaloids, and exposing a developing embryo or fetus to alkaloids from plants, plant products, or plant extracts has shown developmental defects in an animal study [39].

The development of the circulatory system, musculoskeletal system, nervous system, and craniofacial region in 12-day-old rat embryos was assessed using a dissecting microscope. At the middle and high doses of the essential oil, there was a significant retardation of development in the otic system, olfactory system, and branchial bars. This finding is similar to the study conducted in Ethiopia [37] that reported developmental delays in the otic system, olfactory system, branchial bars, and maxillary and mandibular processes in pregnant rats exposed to high doses of methanolic extracts of seeds of *Moringa stenopetala.* This could be because of the presence of alkaloids in *Moringa stenopetala* seed extract and *T. schimperi*, which can cause developmental delays [39]. Another probable explanation would be that *T. schimperi* major phytochemicals such as carvacrol and thymol hinder cellular proliferation and migration [29, 30] which are basic cellular activities for organ system development.

In the current study, external morphological analysis of 20-day-old rat fetuses revealed no apparent anatomical malformations or treatment-related abnormalities in the cranial, nasal, oral, and visceral organs. Consistent with our findings, the previous studies on this plant's acute, subacute, and chronic toxicity were found to be nontoxic [27, 40]. As a result, at doses administered, the *T. schimperi* essential oil has no teratogenic effect on the soft tissue of rat fetuses.

During the late fetal phase, many of the bones in rats ossify. As a result, the level of bone ossification is a significant measure of fetal maturity in developmental toxicity studies [24, 41]. In the current study, the ossification centers of the sternum, vertebrae, hyoid, forelimb, and hind limb bones did not differ significantly between the treatment and control groups on the essential oil of *Thymus schimperi*. The number of ossification centers in the caudal vertebrae and hind limb phalanges was lower at higher doses than the control groups. However, none of them was statistically significant, implying that the test plant does not affect fetal skeletal growth.

In this study, placental weight and fetal weight were significantly decreased. The possible justification for this could be due to *T. schimperi* containing active components such as terpenoids, carvacrol, thymols, o-Cymene, *α*-terpinene, and linalool [42] that can cross the placenta and may affect the placenta and fetal development.

### 4.1. Strengths and Limitations of the Study

All measurements in this study were made by the same examiner to eliminate interobserver errors, which was a study's strength.

There were some limitations to this study. It was a small sample animal study. Animal studies should be followed up with clinical human studies to confirm the findings. Animal studies should use a minimum acceptable number of laboratory animals, which limits the generalizability of the results. Human studies with a larger sample size are needed to assess the effect of *Thymus schimperi* essential oil on embryo and fetal development over long treatment courses. Finally, we are having difficulty finding some up-to-date studies that support this study to use as a reference.

## 5. Conclusion

The results of the developmental toxicity test show that administration of a high dose (260 mg/kg) of the essential oil of *T. schimperi* caused significant delays in fetal and embryonic development, a decrease in the number of implantation sites, and an increase in resorption number, suggesting its developmental toxicity. Similarly, higher doses of *T. schimperi* essential oil resulted in a significant reduction in maternal weight gain, placenta weight, and litter weight. Furthermore, at the middle (130 mg/kg) and high doses (260 mg/kg) of the essential oil extract, there was significant retardation in the development of the otic system, olfactory system, and branchial bars. Therefore, pregnant women should have a general awareness that risks could be associated with taking the essential oil during pregnancy, and consuming too much *T. schimperi* during pregnancy may be harmful.

## Figures and Tables

**Table 1:** Pregnancy outcome following treatment of pregnant rats with the essential oil of *Thymus schimperi*: day 12 experiment.

Parameters	Control groups			Treatment groups	
	G-I (pair-fed control)	G-II (ad libitum)	G-III (EO 65 mg/kg)	G-IV (EO 130 mg/kg)	G-V (EO 260 mg/kg)
Maternal weight gain per group (g)	3.11 ± 0.20	3.10 ± 0.21	3.31 ± 0.50	3.33 ± 0.07	1.73 ± 0.41^**b**^
Implantation sites per litter	10 ± 0.50	10.1 ± 0.71	9.6 ± 0.03	9.1 ± 0.60	8.2 ± 0.55^**b**^
Resorptions per litter	0.40 ± 0.51	0.35 ± 0.45	0.38 ± 0.50	0.42 ± 0.33	1.01 ± 0.61^**b**^

The data are expressed as mean ± SEM, *n* = 10 for each group. ^a^Significant difference compared to the pair-fed control group (*p* < 0.05), ^**b**^significant difference compared to the pair-fed control and *ad libitum* group (*p* < 0.05), and ^c^significant difference compared to the *ad libitum* group (*p* < 0.05). EO: essential oil of *Thymus schimperi*.

**Table 2:** Embryonic growth following administration of the essential oil of *Thymus schimperi* leaves.

Parameters	Control groups		Treatment groups		
	G-I (pair-fed control), *n* = 120	G-II (ad libitum), *n* = 116	G-III (EO 65 mg/kg), *n* = 109	G-IV (EO 130 mg/kg), *n* = 106	G-V (EO 260 mg/kg), *n* = 98
Number of somites/litter	29.2 ± 1.1	30.1 ± 0.8	28.7 ± 1.3	28.1 ± 0.8	26.4 ± 0.9 ^b^
CRL of the embryo (mm)	5.0 ± 0.6	5.1 ± 0.4	4.8 ± 0.5	4.6 ± 0.6	4.3 ± 0.7^b^
Morphological score/litter	46.1 ± 0.2	45.8 ± 0.3	44.7 ± 0.5	44.3 ± 0.4	44.0 ± 0.5^b^

CRL: crown-rump length; results are expressed as mean ± standard deviation of mean; ^b^mean significantly different from pair-fed and *ad libitum* control groups; *p* value is <0.05; *n*: number of embryos; EO: essential oil.

**Table 3:** In vivo development of the rat embryo following treatment with administration of essential oil extract of *Thymus schimperi*: day 12 experiment.

Morphological endpoint	G-I (pair-fed control)	G-II (ad libitum)	G-III (EOT 65 mg/kg)	G-IV (EOT 130 mg/kg)	G-V (EOT 260 mg/kg)
Number of fetus/group	120	116	109	106	98
Yolk sac circulation	3.8 ± 0.51	3.7 ± 0.50	3.60 ± 0.49	3.62 ± 0.50	3.66 ± 0.51
Flexion	2.8 ± 0.33	2.6 ± 0.30	2.7 ± 0.32	2.5 ± 0.31	2.7 ± 0.32
Heart	3.6 ± 0.51	3.6 ± 0.50	3.5 ± 0.49	3.5 ± 0.49	3.6 ± 0.50
Caudal neural tube	4 ± 0.00	4 ± 0.00	4 ± 0.00	4 ± 0.00	4 ± 0.00
Hind brain	3.6 ± 0.50	3.6 ± 0.50	3.5 ± 0.48	3.5 ± 0.48	3.5 ± 0.48
Mid brain	3.56 ± 0.50	3.6 ± 0.50	3.5 ± 0.49	3.4 ± 0.47	3.4 ± 0.47
Fore brain	3.7 ± 0.47	3.7 ± 0.47	3.7 ± 0.47	3.6 ± 0.46	3.6 ± 0.45
Otic system	3.6 ± 0.51	3.5 ± 0.50	3.4 ± 0.55	3.1 ± 0.41^**b**^	3.0 ± 0.40^**b**^
Optic system	3.60 ± 0.50	3.6 ± 0.50	3.5 ± 0.47	3.4 ± 0.46	3.5 ± 0.47
Olfactory system	0.9 ± 0.70	0.8 ± 0.62	0.8 ± 0.62	0.7 ± 0.60^**b**^	0.5 ± 0.42^**b**^
Branchial bars	3.6 ± 0.55	3.5 ± 0.52	3.4 ± 0.50	3.41 ± 0.57	2.9 ± 0.33^**b**^
Maxillary process	1.6 ± 0.54	1.5 ± 0.51	1.4 ± 0.49	1.3 ± 0.48	1.3 ± 0.48
Mandibular process	0.8 ± 0.52	0.7 ± 0.50	0.7 ± 0.50	0.6 ± 0.49	0.6 ± 0.49
Fore limb	2 ± 0.00	2 ± 0.00	2 ± 0.00	2 ± 0.00	2 ± 0.00
Hind limb	2 ± 0.00	2 ± 0.00	2 ± 0.00	2 ± 0.00	2 ± 0.00

Statistical differences between the groups were analyzed by duncan's multiple range tests. Results are expressed as mean ± SDM. ^**b**^Mean significantly different from pair-fed and *ad libitum* control; the *p* value is <0.05.

**Table 4:** Daily food intakes and maternal weight gains of animals in the day 20 administration of essential oil of *Thymus schimperi*.

Groups	Daily food intake (g/day)			Maternal weight gain (g/day)	
	Days 1–5	Days 6–12	Days 13–20	Days 6–12	Days 13–20
G-I (pair-fed control)	15.50 ± 0.16	15.91 ± 0.41	16.51 ± 0.42	5.96 ± 0.61	16.01 ± 0.75
G-II (ad libitum)	15.30 ± 0.07	15.70 ± 0.13	16.45 ± 0.51	5.89 ± 0.72	16.62 ± 0.80
G-III (EO 65 mg/kg)	15.16 ± 0.17	15.53 ± 0.11	16.20 ± 0.22	5.72 ± 0.81	15.70 ± 0.50
G-IV (EO 130 mg/kg)	15.20 ± 0.06	15.66 ± 0.31	16.10 ± 0.09	5.65 ± 0.61	15.65 ± 0.49
G-V (EO 260 mg/kg)	15.37 ± 0.09	15.40 ± 0.05	16.00 ± 0.03	3.32 ± 0.56^**b**^	9.41 ± 0.32^**b**^

Results are stated as mean ± SDM. ^**b**^Results significantly different (*p* < 0.05) from both pair-fed control and ad libitum groups.

**Table 5:** Pregnancy outcomes of the day 20 experiment after administration of *Thymus schimperi* essential oil.

Groups	No. of fetuses	Implantation ns sites	No. of resorptions/litter	No. of live fetuses/dam	No. of dead fetuses/dam
G-I (pair-fed control)	96	9.8 ± 0.74	0.20 ± 0.50	9.40 ± 0.80	0.2 ± 0.03
G-II (ad libitum)	98	10.1 ± 0.89	0.30 ± 0.52	9.50 ± 0.86	0.30 ± 0.04
G-III (EO 65 mg/kg)	86	9.0 ± 0.70	0.40 ± 0.38	8.20 ± 0.74	0.40 ± 0.05
G-IV (EO 130 mg/kg)	82	8.6 ± 1.02	0.40 ± 0.35	7.70 ± 0.72	0.50 ± 0.06
G-V (EO 260 mg/kg)	80	7.7 ± 0.65^**b**^	0.70 ± 0.18^**b**^	7.40 ± 0.71	0.60 ± 0.07

Results are stated as mean ± SDM. ^**b**^results significantly different (*p* < 0.05) from both pair-fed control and *ad libitum* groups.

**Table 6:** Mean fetal growth following treatment with essential oil of *Thymus schimperi* in the day 20 experiment.

Groups	Fetal growth		
	Litter weight/fetus (g)	CRL/fetus (cm)	Placental weight/fetus (g)
G-I (pair-fed control)	3.41 ± 0.09	3.18 ± 0.11	0.55 ± 0.05
G-II (ad libitum)	3.38 ± 0.10	3.18 ± 0.11	0.57 ± 0.06
G-III (EO 65 mg/kg)	3.34 ± 0.09	3.13 ± 0.15	0.55 ± 0.06
G-IV (EO 130 mg/kg)	3.25 ± 0.12	3.08 ± 0.13	0.55 ± 0.06
G-V (EO 260 mg/kg)	2.71 ± 0.11^**b**^	2.91 ± 0.18^**b**^	0.48 ± 0.05^**b**^

Results are stated as mean ± SDM. ^**b**^Results significantly different (*p* < 0.05) from both pair-fed control and ad libitum groups.

**Table 7:** External malformations of rat fetuses after administration of the essential oil of *Thymus schimperi*.

Group	Fetus	Observed malformations (%)							
	Examined	AC	EC	SB	SC	KY	LD	MT	AEG
G-I (pair-fed control)	96	0	0	0	0	0	0	0	0
G-II (ad libitum)	98	0	0	0	0	0	0	0	0
G-III (CT 500 mg/kg)	86	0	0	0	0	0	0	0	0
G-IV (CT 1000 mg/kg)	82	0	0	0	0	0	0	0	0
G-V (CT 2000 mg/kg)	80	0	0	0	0	0	0	0	0

The percentage of malformations is represented as a percentage of the total number of malformations (chi-square). AC: anencephaly, EC: exencephaly, SB: spina bifida, KY; kyphosis, SC: scoliosis, LD: limb defect, MT: missed tail, and AEG: agenesis of external genitalia.

**Table 8:** Visceral malformations of rat fetuses after administration of the essential oil of *Thymus schimperi*.

Group	Fetus	Observed malformations (%)										
	Examined	HC	MO	AO	CP	NSD	REAA	VSD	DH	RA	HU	CT
G-I (pair-fed control)	96	0	0	0	0	0	0	0	0	0	0	0
G-II (ad libitum)	98	0	0	0	0	0	0	0	0	0	0	0
G-III (CT 500 mg/kg)	86	0	0	0	0	0	0	0	0	0	0	0
G-IV (CT 1000 mg/kg)	82	0	0	0	0	0	0	0	0	0	0	0
G-V (CT 2000 mg/kg)	80	0	0	0	0	0	0	0	0	0	0	0

Results are expressed as a percentage of malformations (chi-square). HC: hydrocephalus, MO: microphthalmia, AO: anophthalmia, CP: cleft palate, NSD: nasal septal defect, REAA: retroesophageal aortic arch, VSD: ventricular septal defects, DH: diaphragmatic hernia, RA: renal agenesis, HU: hydroureters, and CT: cryptorchid testes.

**Table 9:** Skeletal malformations of a 20-day-old rat fetuses following treatment with the essential oil of *Thymus schimperi*.

Group	Percentage of skeletal malformations				
	Sternum^**a**^	Hyoid^**b**^	Ribs^**c**^	Thoracic vertebrae^**d**^	Caudal vertebrae^**e**^
G-I (pair-fed control), *n* = 50	12	0	0	0	8
G-II (ad libitum), *n* = 50	14	0	0	0	10
G-III (EO 65 mg/kg), *n* = 50	16	0	0	0	12
G-IV (EO 130 mg/kg), *n* = 50	18	0	0	0	14
G-V (EO 260 mg/kg), *n* = 50	18	0	0	0	16

The percentage of skeletal malformations is calculated (chi-square). ^**a**^<4 ossification centers on the sternum; ^**b**^no ossification signs on the hyoid bone; ^**c**^no ossification signs on the ribs; ^**d**^<13 ossification centers on the thoracic centra; ^**e**^<4 ossification centers on the caudal vertebrae.

**Table 10:** Skeletal malformations of a 20-day-old rat fetuses following treatment with the essential oil of *Thymus schimperi*.

Group	Percentage of skeletal malformations of limb bones			
	Metacarpus^**a**^	Metatarsal^**b**^	Forelimb phalanges^**c**^	Hindlimb phalanges^**d**^
G-I (pair-fed control), *n* = 50	6	4	10	16
G-II (ad libitum), *n* = 50	4	4	12	16
G-III (EO 65 mg/kg), *n* = 50	6	6	12	20
G-IV (EO 130 mg/kg), *n* = 50	8	6	14	20
G-V (EO 260 mg/kg), *n* = 50	8	6	14	26

The number of skeletal malformations is expressed as percentage (chi-square). ^**a**^<3 metacarpus; ^**b**^<3 metatarsus; ^**c**^no forelimb proximal phalanges; ^**d**^no hindlimb proximal phalanges.

## Data Availability

Upon request to the corresponding author, the experimental data used to support the results of this study will be made available.
